# Antioxidant, Phenolic, Flavonoid, and Mineral Content of *L. officinalis* and Its Cytotoxic Effect on Human Embryonic Kidney (Hek‐293) Cells

**DOI:** 10.1002/fsn3.4608

**Published:** 2025-03-19

**Authors:** Yeliz Kaya Kartal, Derya Ozalp Unal, Halil Ibrahim Ozkan, Adnan Adil Hismiogullari, Tevhide Sel

**Affiliations:** ^1^ Ankara University Faculty of Veterinary Medicine Department of Biochemistry Ankara Turkey; ^2^ Field Crops Central Research Institute Directorate Ankara Turkey; ^3^ Atatürk University Faculty of Medicine Department of Medical Biochemistry Erzurum Turkey; ^4^ Balıkesir University Faculty of Medicine Department of Medical Biochemistry Balıkesir Turkey

**Keywords:** antioxidant, cell culture, cytotoxicity, phenols

## Abstract

Cherry laurel ( *L. officinalis* ) is a well known natural product and folk medicine in the Black Sea region of Turkey. The aim of this study was to investigate the antioxidant effect, polyphenolic, and mineral content of cherry laurel and the cytotoxic effect of its methanolic extraction on human embryonic kidney cells. The total phenolic content of *L. officinalis* was found to be 1.28 mg GAE/g, while the flavonoid content was 1.26 mg RE/g. The DPPH scavenging activity was 118.76 μg/g. Total antioxidant capacity was found to be 3.54 mM/100 g and in HPLC analysis only chlorogenic acid (101 μg/g) could be detected, but cyanidin‐3‐glucoside chloride, resveratrol, vanillic acid, (+)‐catechin, and (−)‐epicatechin could not. The highest mineral content was found in magnesium levels (46.10 ± 0.57 μg/g), but also contained selenium (9.90 ± 0.78 μg/g), silver (4.46 ± 0.27 μg/g), lead (1.34 ± 0.08 μg/g), zinc (1.31 ± 0.11 μg/g), and copper (0.66 ± 0.05 μg/g). Trace amounts of manganese (0.17 ± 0.02 μg/g) and mercury (0.08 ± 0.01 μg/g) were found in aqueous extraction of *L. officinalis* but in ethanolic and methanolic extractions these elements could not be detected. In all elements there was a statistically significant increase in water extraction of *L. officinalis* . Cobalt could not be detected in any of the extractions. The IC_50_ concentration of *L. officinalis* on Hek‐293 cells was found to be 370 mg/mL. As a conclusion, *L. officinalis* is rich in chlorogenic acid and is a good antioxidant fruit. The high antioxidant activity, phenolic and flavonoid content, and mineral content are mostly used to decrease oxidative stress; however, it should not be forgotten that antioxidants may also have pro‐oxidant effects and should be investigated more on healthy and unhealthy cells.

## Graphical abstract

*L. officinalis* is mostly known as a hypoglycemic summer fruit and is used as a folk medicine in Turkey between diabetic patients. Its phenolic, flavonoid, mineral content (mostly Mg), antioxidant capacity is high, and the phenolic compound found in cherry laurel due to HPLC analysis is mostly chlorogenic acid. These contents are good sources for preventing chronic diseases, but in this case, even in healthy cells, natural products have shown cytotoxic effects in a dose‐dependent manner.

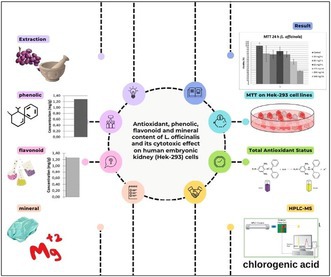

## 1 Introduction

Cherry laurel is a red and purple summer fruit belonging to the *Laurocerasus officinalis* genus, Angiospermae subdivision of the Spermatophyta section, Dicotyledonae class, Rosaceae family, Prunoideae subfamily, and is grown in the Black Sea region of Turkey, especially in the Trabzon, Ordu, and Samsun regions. It also grows in the Balkans, Northern Ireland, Western Europe, Southern and Western Caucasus, Iran, Eastern Marmara, and some Mediterranean countries. It can generally be grown in clayey–sandy soils and humid environments in temperate climates. The nutritional content of *L. officinalis* is very rich, especially in terms of Ca, Mg, and P. Its carbohydrate content is higher than its protein and fat contents. The water content is quite high. Apart from all these, Na, K, and Fe are also present in small amounts. It is rich in lutein and beta carotene. In short, it is a very nutritious and useful fruit and displays high antioxidant content (Damlaci 2009; Karaman and Elgin Cebe 2016; Karataş and Uçar 2018).

Considering the antioxidant effect of the plant, it was found to be rich in phenolic acid and flavonoids, according to HPLC analysis. Gallic acid, protocatechuic acid, p‐hydroxy benzoic acid, chlorogenic acid, vanillic acid, and coumaric acid have been found in *L. officinalis* species. It has been observed that catechin and anthocyanins are present in cherry laurel fruits, too. As a result of different phytochemical tests, tannins, alpha tocopherols, sterols, and starch have also been reported in the leaves of the fruit. Phenolic acids and carboxylic acids were found in studies conducted in the fruit parts of the plant, and important fatty acids were found in the seed parts (Damlaci 2009; Karahalil and Şahin 2011).

Polyphenols and flavonoids can protect against oxidative stress due to their high antioxidant effect. Vegetables and fruits naturally contain these antioxidants. Polyphenolic compounds can show their antioxidant effect by scavenging the reactive oxygen species (ROS), chelating the metal ions, or being enzyme modulators (Pietta et al. 1998; Dugas et al. 2000). For example, quercetin is known as an intracellular iron chelator (Ferrali et al. 1997) and resveratrol, which is a polyphenol, can inhibit the activity of cyclooxygenase‐2 (COX‐2), a pathway responsible for cancer progression (Subbaramaiah et al. 1998).

Oxidative stress is mainly the imbalance of production of the reactive oxygen species and antioxidants. In physiological conditions, free radicals are not eliminated because their concentration is low. But the consumption of polyphenols in high concentrations can also cause damage to health. (Pereira Lima et al. 2014). Also, flavonoids, in high doses, can have pro‐oxidant effects and result in the production of free radicals. On the other hand, high doses of flavonoids can inhibit the key enzymes of some important hormones (Skibola and Smith 2000). So, the intake of vegetables and fruits with high polyphenolic compounds should be incorporated in a planned way in diets.

The aim of this research was to identify the polyphenolic and flavonoid content, the DPPH scavenging activity, total antioxidant capacity and HPLC and mineral content analysis of *L. officinalis* fruit extraction obtained from Bartin and Kastamonu, cities in the North‐west region of Turkey. The second aim of the research was to investigate the cytotoxic effect of *L. officinalis* on Hek‐293 cells.

## 2 Materials and Methods

### 2.1 Chemicals

2,2‐Diphenyl‐1‐picrylhydrazyl (Sigma, cat no. D9132‐1G), AlCl_3_.6H_2_O (Sigma, cat no. A0718‐500G), gallic acid (Sigma, cat no. G7384‐100G), rutin trihydrate (Alfa Aesar, lot no. 10181593), NaNO_2_ (Merck, cat no. 0090815), and Na_2_CO_3_ (Merck, cat no. S033173) were obtained for the content analysis, and DMEM (Gibco, cat no. 41966029), gentamicin (Sigma, cat no. G1264‐50MG), HEK‐293 cell line (ATCC, CRL‐1573), MTT (Sigma, cat no. M2128‐1G), and sodium dodecyl sulfate (Sigma, cat no. L3771‐500G) were obtained for cell culture.

### 2.2 Supply of *L. officinalis*

Cherry laurel fruits were purchased fresh from a market in Istanbul/Pendik in September, the harvest time in 2019. The market obtained the fruits from Bartin and Kastamonu provinces, which are located in the west of Black Sea region. The seed was separated from fruit and fruits were protected at −20°C until analysis.

### 2.3 Extraction of *L. officinalis*

The extraction method of Agcam and Akyildiz (Agcam and Akyildiz 2015) was modified, and a methanolic extraction solution was prepared according to Bronnum‐Hansen, Jacobsen, and Flink (1985). (methanol: 0.1 N HCl, 85:15, v/v). Cherry laurel fruits were extracted with the prepared extraction solution. 5 g of wet fruit was added to a 50 mL extraction solution. The fruit (5 g) was fully crushed in a mortar, and the first 25 mL extraction solution was added and incubated for 20 min in room temperature. After the incubation time, it was mixed with the help of vortex and was centrifuged in 4000 rpm for 10 min. The supernatant of the mix was collected. This process was done twice. The methanolic extraction method was used for the determination of polyphenol, flavonoid, and DPPH scavenging activity analysis. For HPLC content analysis, only methanol (without HCl) was used for the extraction (0.5 g wet fruit/40 mL methanol). For the mineral content analysis, all three (methanol: 0.1 N HCl, ethanol: 0.1 N HCl, and distilled water: 0.1 N citric acid) extractions were used according to the methods described, and the results were compared. To apply the extracted fruit to cell culture, the methanolic extraction was evaporated and dissolved in DMSO (1%) and diluted with the culture media.

### 2.4 Polyphenol Content Analysis

The modified method of Singleton and Rossi (Singleton and Rossi 1965) was used to determine the phenolic compound. Gallic acid was used as a standard; this method is known as the folin ciocalteu method too.

### 2.5 Total Flavonoid Content Analysis

The method of Boateng et al. (2008) was modified. Rutin trihydrate was used for the standart curve.

### 2.6 DPPH Scavenging Activity

In this analysis, the modified method of Shirazi et al. (2014) was used. As a standard, gallic acid was used. Oxidized DPPH^˙^ gives a dark purple color in methanol. An antioxidant compound gives an electron to oxidized DPPH^˙^, and the antioxidant compound will decrease. The purple color turns yellow with the increase of antioxidant compounds (Deng et al. 2011; Sherer and Godoy 2009).

### 2.7 Total Antioxidant Capacity of *L. officinalis*

This works on the principle of transforming the dark blue–green colored ABTS radical into the colorless reduced ABTS form, and the results were obtained by reading the ODs at 660 nm (Erel 2004). The analysis was done with the kit obtained from Relassay (Turkey, cat no. RL0017) TAC kit.

### 2.8 HPLC Content Analysis

HPLC content analysis was carried out at Bezmialem University Pharmaceutical Application and Research Center. *L. officinalis* extract was analyzed on LC–HRMS device. HPLC and MS conditions of the method are given in Table 1. As standard chlorogenic acid, cyanidin‐3‐glucoside chloride, resveratrol, vanillic acid, (+)‐catechin, and (−)‐epicatechin were used.

**TABLE 1:** HPLC–MS conditions.

		HPLC conditions	
Mobile phase A	Formic acid (%1)—H_2_O		
Mobile phase B	Formic acid (%1)—MeOH		
Colon	Troyasil C18 HS	150 × 3 mm	3.5 μm
Gradient	Time	Flow rate (mL/dk)	%B
	0.00	0.35	50
	1.00	0.35	50
	3.00	0.35	100
	6.00	0.35	100
	7.00	0.35	50
	15.00	0.35	50

		MS conditions (mass spectrometry)	
System	Thermo ORBİTRAP Q‐EXACTİVE		
Ion source	ESİ		
Mass scan range	100–900 m/z		
Gas flow rate	45		
Helper gas flow rate	10		
Spray voltage (kV)	3.80		
Capillary temperature (°C)	320		
S‐lens RF stage	50.0		

		Environmental conditions	
Temperature	22.0°C ± 5.0°C		
Relative humidity	50% ± 15% rh		

### 2.9 Mineral Content of *L. officinalis*

All samples were properly thawed on the study day, and the element levels were measured by an ICP–MS device. The principle of this device is that the samples in solution are sent to the ionization unit with argon (Ar) gas, and the atoms that ionized at high temperature are separated and analyzed in the mass spectrometer. For this, the samples were first ground in the microwave oven. For each sample, 0.1 mL of extracted samples were taken, and 2 mL of 65% HNO_3_ was added, followed by 0.25 mL of 30% H_2_O_2_. This was incubated for 15–20 min and then burned in a microwave oven at 180°C for 20 min. Standard solutions for the elements to be analyzed (Al, Cr, Mn, Fe, Co, Ni, Cu, Zn, As, Se, Ag, Cd, and Pb) were prepared using 2% nitric acid solution at increasing concentrations. Calibration curves were drawn. Indium, scandium, germanium, and bismuth were used as internal standards to correct for deviations in the calibration curve during analysis. The samples diluted 1/10 times were subjected to elemental analysis in an inductively coupled plasma mass spectrometer (ICP‐MS, Agilent 7700).

### 2.10 Preparation of HEK‐293 Cell Lines

Cell lines were incubated in CO_2_ (5%) incubators and at 37°C. Before incubation, cell lines were poured into a T75 cm^3^ flask, and to increase the amount of the cell, 10% of fetal bovine serum in DMEM with 1% of nonessential aminoacid was used. To protect the cell from infection, the medium contained penicillin–streptomycin and gentamycin.

### 2.11 Methylthiazole Diphenyl Tetrazolium (MTT) Analysis

Rapid colorimetric assay based on the cleavage of the tetrazolium ring of MTT (3‐(4,5‐dimethylthazol‐2‐yl)‐2,5‐diphenyl tetrazolium bromide) by dehydrogenases in active mitochondria of living cells as an estimate of viable cell number (van Merloo et al. 2011). With this analysis, the IC_50_ concentration of *L. officinalis* in the HEK‐293 cell line was found.

### 2.12 Statistical Analysis

Statistical analysis was done in the SPSS 21.0 package program. In mineral content of *L. officinalis*, three different extractions were used, and to see the significant differences between extractions, ANOVA was applied for concentrations that met the normality assumption, and a Kruskal Wallis test was applied for concentrations that did not. The Tukey test was applied for pairwise comparisons and *p* < 0.05 was accepted as statistically significant.

## 3 Results

### 3.1 Polyphenolic, Total Flavonoid Content, and DPPH Inhibition Activity of *L. officinalis*

**FIGURE 1:**
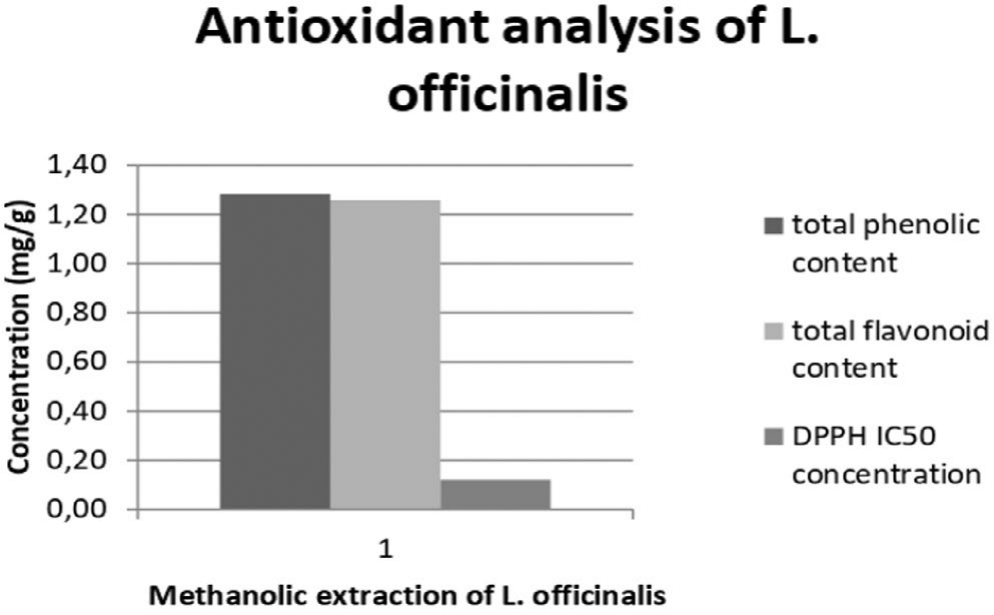
Phenolic and flavonoid content and DPPH scavenger activity of *L. officinalis* (mg/g fruit).

The total phenolic content of *L. officinalis* was found to be 1.28 mg GAE per gram and the total flavonoid content of *L. officinalis* was 1.26 mg RE per gram. Comparing the phenolic and flavonoid content of *L. officinalis* fruit, it can be said that 98% of the fruit contains flavonoids. At least 118.76 μg (0.118 mg) GAE of per gram fruit can inhibit 50% of DPPH (or the result as μg/mL is 11.88). In Figure 1, the concentrations of antioxidant analysis are given as a graph.

### 3.2 Total Antioxidant Capacity (TAC) of L. Officimalis

Three different methanolic extractions of *L. officinalis* were performed. Results of TAC are given in Table 2 as mean and min–max values. The concentration of *L. officinalis* extraction was found at 3.54 mM/100 g. The minimum TAC was 2.66 mM/100 g, and the maximum concentration was 4.12 mM/100 g.

**TABLE 2:** TAC concentration of *L. officinalis* extract.

	*n*	Concentration (mean)	Min–max
Total antioxidant capacity	3	3.54 mM/100 g	2.66–4.12 mM/100 g

### 3.3 HPLC Content Analysis of *L. officinalis*

Phenolic component determination of *L. officinalis* extract was determined by HPLC‐MS, and it was found that it did not have cyanidin‐3‐glycoside chloride, resveratrol, vanillic acid, (+)‐catechin, and (−)‐epicatechin content, but it had 101 μg of chlorogenic acid in per gram of *L. officinalis* extract (or 10.1 mg/100 g wet fruit). Table 3 shows the table of the analysis result report.

**TABLE 3:** Polyphenolic content analysis results of HPLC‐MS.

	Name of compound	*L. officinalis* (mg/L)	Relative uncertainty (%) mg/L	*L. officinalis* content (μg/g)
1	Chlorogenic acid	0.028	3.58	101
2	Vanillic acid	0	3.49	0
3	Resveratrol	0	3.17	0
4	(+)‐Catechin	0	3.31	0
5	(−)‐Epicatechin	0	3.17	0
6	Cyanidin‐3‐glucoside chloride	0	2.11	0

### 3.4 Mineral Content of *L. officinalis*

The mineral content of *L. officinalis* extraction was analyzed with ICP‐MS and lead (Pb), mercury (Hg), silver (Ag), zinc (Zn), selenium (Se), magnesium (Mg), copper (Cu), mangane (Mn), and cobalt (Co) concentrations were measured. In this analysis, three methanolic, three ethanolic, and three water extraction of *L. officinalis* was used. In Table 4, the mineral content as Me ± SE are given for all extraction methods.

**TABLE 4:** Mineral content of *L. officinalis* (a: the lowest concentration, b: highest concentration, ab: no significant changes, c: all extractions have statistical significant differences with each other and here the highest concentration is showed with c, there is no statistical significance in those not lettered with a superscript).

	*n*	Ethanolic extraction (Me ± SE)	Methanolic extraction (Me ± SE)	Water extraction (Me ± SE)
Pb (μg/g)	3	0.43 ± 0.03^ab^	0.27 ± 0.02^a^	1.34 ± 0.08^b^
Hg (μg/g)	3	< 0.00^a^	< 0.00^a^	0.08 ± 0.01^b^
Ag (μg/g)	3	1.33 ± 0.20^a^	2.82 ± 0.08^b^	4.46 + 0.27^c^
Zn (μg/g)	3	0.52 ± 0.02^a^	0.68 ± 0.03^ab^	1.31 ± 0.11^b^
Se (μg/g)	3	0.83 ± 0.04^a^	6.80 ± 0.30^b^	9.90 ± 0.78^c^
Mg (μg/g)	3	14.67 ± 0.35^a^	26.90 ± 0.96^b^	46.10 ± 0.57^c^
Cu (μg/g)	3	0.23 ± 0.01^a^	0.42 ± 0.02^ab^	0.66 ± 0.05^b^
Mn (μg/g)	3	< 0.00^a^	< 0.00^a^	0.17 ± 0.02^b^
Co (μg/g)	3	< 0.00	< 0.00	< 0.00

*L. officinalis* water extraction contains more mineral compounds than the methanolic or ethanolic extractions. The fruit extract does not contain any cobalt mineral in all extraction procedures and mercury and mangane elements could be analyzed only in water extractions.

By comparing the results with extraction methods, it can be said that except cobalt, in all elements, there was a statistically significant increase in water extraction of *L. officinalis* . In silver, selenium, and magnesium contents, a significant increase was found in methanolic extraction compared with ethanol.

### 3.5 IC_50_ Concentration of *L. officinalis*

**FIGURE 2:**
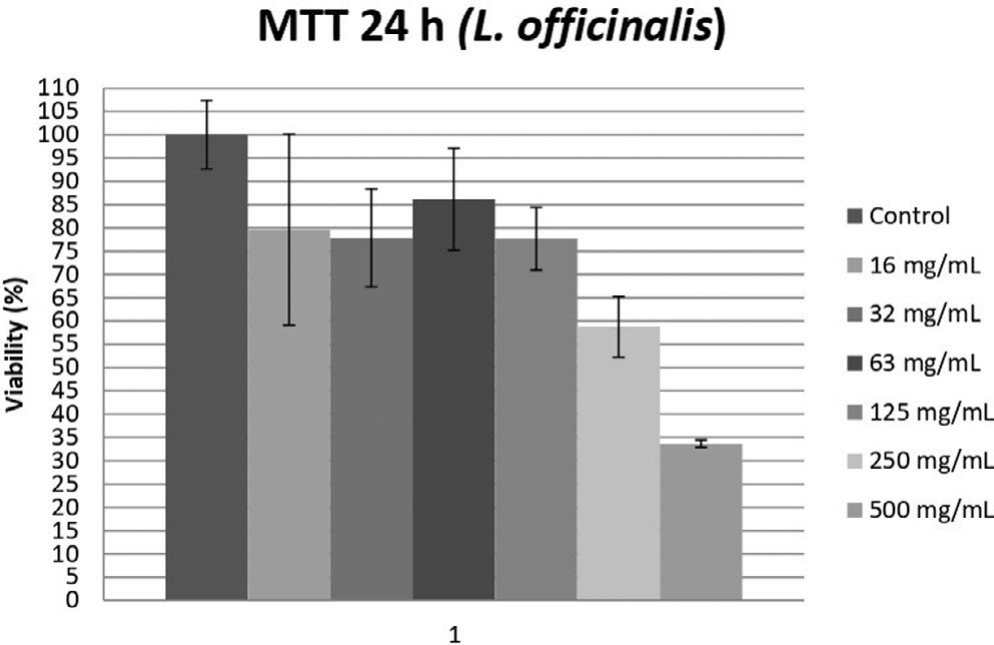
The MTT results of various concentrations of *L. officinalis* (Me ± SD).

The IC_50_ concentration of *L. officinalis* was found 370 mg/mL. The viability of HEK‐293 can be seen in Figure 2.

The doses of *L. officinalis* were prepared as 0 mg/mL (control), 15.63, 31.25, 62.50, 125.00, 250.00, and 500.00 mg/mL. The 0 mg/mL dose was accepted as 100% viability of HEK‐293. Application of *L. officinalis* on HEK‐293 in various doses decreased the viability to 79.61%, 77.85%, 86.14%, 77.70%, 58.74%, and 33.66%.

## 4 Discussion

The total phenolic compound of methanolic extraction of *L. officinalis,* according to the research of Karahalil and Sahin (Karahalil and Şahin 2011), was found to be 1.09 mg GAE/100 g dry weight, and the total flavonoid concentration was found to be 0.08 mg QE/100 g dry weight. The samples were collected from Trabzon province, which is located in the east side of black sea region. Another study with *L. officinalis* supplied from Giresun province analyzed the phenolic content in fresh weight. The phenolic content was found at 454 mg GAE/100 g. Compared with these two studies, the result of our research does not contain as much as the study of Alasalvar, Al‐Farsi, and Shahidi (2005). The reason for this differences could be the region the fruits were sampled from. But especially, dry weight and fresh weight phenolic content can differ from each other. In order to see the difference of dry weight, a study conducted by Demir et al. 2017 investigated the cytotoxic effect of *L. officinalis* in various human cancer cells and they analyzed the polyphenolic content of dry *L. officinalis* samples and extracted it with dimethylsulfoxide (DMSO) and found 33.7 mg GAE per g sample. Annother study used different genotypes of *L. officinalis* and found the total phenolic content between 24 and 75 mg per g dry weight (Halilova and Ercisli 2010). This difference could be because of the extraction method (Lou et al. 2016) they used, and dry weight and fresh weight (Tiwari et al. 2005) fruit can change the results of contents.

Comparing the flavonoid contents of Karahalil and Sahin (Karahalil and Şahin 2011) and our study, Karahalil and Sahin found only about 7% of total flavonoid in all polyphenol content, while in our study it was found to be 98%. This could be due to the region and climate. A study done with fresh dried and grounded *L. officinalis* ethanolic extraction fruits gave 0.08 mg GAE/g polyphenol content and 0,003 mg QE/g flavonoid content, which means that the 26% of phenolic campounds are present as flavonoids (Cirrik et al. 2024). The results were too low compared with our study but the reason could be the extraction solution because in some studies ethanolic extractions gave a lower concentration in content analysis of phenolic compounds compared to methanolic extractions (Ozalp and Sel 2024). The study of Kolayli et al. (2003), used the water soluble *L. officinalis* extract and found only 10.4 mg GAE/100 g in phenolic content analysis. So, all solvents can change the results of content analysis (Siddhuraju and Becker 2003).

The DPPH scavenge activity results were found to be 28.6 μg/mL (Kolayli et al. 2003) in methanolic extract and 20 mg/mL (Cirrik et al. 2024) in ethanolic extraction. These concentrations are the results for scavenging the 50% of DPPH. A study conducted by Beyhan, Demir, and Yurt (2018) researched the contents of various different genotypes of *L. officinalis* and found the DPPH scavenge activity between 3% to 25% for 3 mg/mL concentration in dry fruit. So this means that it can show differences between genotypes (Beyhan et al. 2018). In our study, the IC_50_ concentration of wet fruit was found 11.88 μg/mL. The lower the IC_50_ concentration, the better scavenging activity. These differences are not just because of the extraction methods or solvents that are used. The genotype, climate, soil structure, irrigation (receiving rain), region, or province (Kamenetsky and Okubo 2012), etc. can affect the changes.

The phenolic–flavonoid content analysis by using the HPLC method is more specific but an expensive method. Karahalil and Sahin (Karahalil and Şahin 2011) analyzed 33 mg chlorogenic acid, 7.69 mg vanillic acid, 3.40 mg catechin in 100 g dry weight and epicatechin was not detected in HPLC analysis. The study of Alasalvar, Al‐Farsi, and Shahidi (2005) found 102.64 mg chlorogenic acid and 10.48 mg vanillic acid in 100 g of fresh weight in HPLC content analysis. But in our study only chlorogenic acid could be detected and the result was found 10.1 mg/100 g fresh weight. The first given study about HPLC used the samples of Trabzon province and the second used samples from Giresun province, two of the provinces are located in east side of black sea region which receives much more rain than the Bartın and Zonguldak provinces, located in the west side of black sea region. But still, the contents of two near located provinces are different from each other. This time the harvest time, year, the rain, received that year, genotypes (Siddhuraju and Becker 2003), the difference of being dry or wet weight (Tiwari et al. 2005) and the extraction method (Lou et al. 2016) etc., all of these reasons can be the explanation of that variety. The polyphenolic content and type of phenols due to HPLC analysis are important because the active ingredients of foods gave us the antioxidant power on cells too. In a study the cytotoxic effect of chlorogenic acid was studied on Caco‐2 and HT‐29 MTX cells. It was found that the IC_50_ value of chlorogenic acid on Caco‐2 cells was approximately 75 and 190 μg/mL on HT‐29 MTX cells (Volstatova et al. 2019). This means that HT‐29 MTX cells are more resistant to antioxidants.

To extract the phenolic compounds, mostly the best solvent is methanol because of its high polarity to the phenolic compounds, as the phenolic compound can dissolve better in methanol (Siddhuraju and Becker 2003). This was the reason methanolic extraction of *L. officinalis* was used to analyze the phenolic and flavonoid contents and the antioxidant activity of the fruit. But in mineral content analysis, in order to observe the difference of water and organic solvents, three different extractions of *L. officinalis* were preferred (methanol, ethanol, and water).

In a study, the water extraction of *L. officinalis* was done and mineral contents were analyzed. It was found that the water extraction of *L. officinalis* contains 179 mg/kg Mg, 0.8 mg/kg Cu, 1.9 mg/kg Zn and 24.2 mg/kg Mn. Co and Pb elements were below the detection limits so it could not be detected (Kolayli et al. 2003). In an another study 12 different *L. officinalis* fruits from same province were collected and the mineral contents were compared. The Mg content differ between 14 and 22 mg/100 g fresh weight, the concentration of Mn was found between 1 and 3.1 mg/100 g, Cu was found 0.1 to 0.3 mg/100 g and at least the Zn concentrations were found between 0.1 and 0.4 mg/100 g (Esringu et al. 2016). In an other study with *L. officinalis* , Mg content was 10.45 mg/g, Cu was 11.99 mg/g, Zn was 3.75 mg/g, Co was 0.06 mg/g, and Hg, Pb, and Ag could not be detected in the fruits sampled from Trabzon (Eken et al. 2017). Comparing the results of this three researches with our study, Mg content is the highest in all studies and the study of Kolaylı et al. and ours are closer to each other than the other studies but the Mg content is much higher than ours. In all extractions except ours HNO_3_ was used before the water was added. But in our study, water and citric acid was used for the extraction. The differences of mineral contents can be explained like the reasons in phenolic compounds. So rain, soil, genotype, province, climate, extraction method etc. can affect the contents of minerals.

Normally, natural products, because of their high polyphenol contents and antioxidative effects, are widely used in cancer or other diseases. The high antioxidant properties of natural products make them good sources to fight or to protect from diseases (Marino et al. 2023). A study used several human cancer cell lines and normal foreskin fibroblast cell line and investigated the cytotoxic effect of *L. officinalis* on these cells. The IC_50_ values of prostate adenocarcinoma (PC‐3) and breast adenocarcinoma (MCF‐7) could not be calculated, and the results were given as higher than 500 μg/mL. The IC_50_ concentration for colon adenocarcinoma (WiDr) was 265.2 μg/mL, for lung carcinoma (A549) 314.5 μg/mL, for hepatocellular carcinoma (HepG2) 357.5 μg/mL, for cervix adenocarcinoma (HeLa) 396.1 μg/mL, and for fibroblast 359.1 μg/mL was found. Although its cytotoxic effect on cancer cells is more promising for colon adenocarcinoma than other cancer cells, it kills fibroblast cells earlier than cervix adenocarcinoma cells, so it shows that it has also cytotoxic effect on healthy cells in a certain overdose (Demir et al. 2017).

The cytoproliferative effect of tannic acid (TA) on Hek‐293 cell lines was investigated, and it was found that TA increased the viability of Hek‐293 cells in a 24 h treatment. The IC_50_ value of TA was found to be 8.9 μM but after 300 μM treatment the cell viability began to increase in a dose dependent manner (Perumal et al. 2019). In our study, only the cytotoxic effect, so the IC_50_ value was found, and it was 370 mg/mL. Comparing with fibroblast cells (Demir et al. 2017), a 1000 time more concentration is needed for the IC_50_ value of Hek‐293 cells. However, in order to find the cytoproliferative effect doses more than 500 mg/mL have to be applied on the cells.

## 5 Conclusions

Content of natural products varies due to many genetic and environmental effects. In most articles, the mineral content analysis of natural compounds uses the water extraction method. It can be said that minerals dissolve better in water so, using water extraction compared with ethanol or methanol could be preferred. But in most cases, natural products are extracted with organic compounds like ethanol, methanol, or sometimes acetone. After the extraction, the products are applied on cell lines or are used in animal studies. So to know the mineral content given to cell lines or animals, it could be better to analyze the mineral content of other extraction methods too.

The use of natural products in cancer cell lines has been widely reported. Mostly, the cytotoxic effect of natural products is thought to be due to the beneficial antioxidant substances they contain (polyphenols and minerals). But overdoses of antioxidants can have pro‐oxidant effects, which are cytotoxic for normal cells but cytoproliferative for cancer cells. So the usage of natural products in normal cells has to be investigated more, and according to the results, products can be applied to cancer cells. Further research is needed to see the effect of *L. officinalis* in normal and cancer cells. To know how it affects the cells, more analysis, like the inflammatory pathways or cancer pathways needs to be investigated.

## Author Contributions

**Yeliz Kaya Kartal:** data curation (lead), formal analysis (lead), investigation (lead), methodology (lead), project administration (lead), writing – original draft (lead), writing – review and editing (lead). **Derya Ozalp Unal:** investigation (supporting), methodology (supporting), resources (supporting). **Halil Ibrahim Ozkan:** data curation (supporting), methodology (supporting), validation (supporting). **Adnan Adil Hismiogullari:** methodology (supporting), supervision (supporting), writing – original draft (supporting), writing – review and editing (supporting). **Tevhide Sel:** formal analysis (equal), investigation (equal), methodology (equal), supervision (lead).

## Conflicts of Interest

The authors declare no conflicts of interest.

## Data Availability

The data that support the findings of this study are available from the corresponding author upon reasonable request. The data are not publicly available due to privacy or ethical restrictions.
